# The non-canonical inflammasome activators Caspase-4 and Caspase-5 are differentially regulated during immunosuppression-associated organ damage

**DOI:** 10.3389/fimmu.2023.1239474

**Published:** 2023-12-01

**Authors:** Mohamed Ghait, Shivalee N. Duduskar, Michael Rooney, Norman Häfner, Laura Reng, Bianca Göhrig, Philipp A. Reuken, Frank Bloos, Michael Bauer, Christoph Sponholz, Tony Bruns, Ignacio Rubio

**Affiliations:** ^1^ Integrated Research and Treatment Center, Center for Sepsis Control and Care, Jena University Hospital, Jena, Germany; ^2^ Department of Internal Medicine IV, Jena University Hospital, Jena, Germany; ^3^ Department of Gynecology, Jena University Hospital, Jena, Germany; ^4^ Department for Anesthesiology & Intensive Care Medicine, Jena University Hospital, Jena, Germany; ^5^ Department of Medicine III, University Hospital RWTH Aachen, Aachen, Germany

**Keywords:** CASP4, CASP5, NLRP3, sepsis, liver failure, pyroptosis

## Abstract

The non-canonical inflammasome, which includes caspase-11 in mice and caspase-4 and caspase-5 in humans, is upregulated during inflammatory processes and activated in response to bacterial infections to carry out pyroptosis. Inadequate activity of the inflammasome has been associated with states of immunosuppression and immunopathological organ damage. However, the regulation of the receptors caspase-4 and caspase-5 during severe states of immunosuppression is largely not understood. We report that *CASP4* and *CASP5* are differentially regulated during acute-on-chronic liver failure and sepsis-associated immunosuppression, suggesting non-redundant functions in the inflammasome response to infection. While *CASP5* remained upregulated and cleaved p20-GSDMD could be detected in sera from critically ill patients, *CASP4* was downregulated in critically ill patients who exhibited features of immunosuppression and organ failure. Mechanistically, downregulation of *CASP4* correlated with decreased gasdermin D levels and impaired interferon signaling, as reflected by decreased activity of the *CASP4* transcriptional activators IRF1 and IRF2. Caspase-4 gene and protein expression inversely correlated with markers of organ dysfunction, including MELD and SOFA scores, and with GSDMD activity, illustrating the association of *CASP4* levels with disease severity. Our results document the selective downregulation of the non-canonical inflammasome activator caspase-4 in the context of sepsis-associated immunosuppression and organ damage and provide new insights for the development of biomarkers or novel immunomodulatory therapies for the treatment of severe infections.

## Introduction

A dysregulated host response to infection can lead to sepsis, a life-threatening condition that culminates in organ failure (1). Typically, sepsis-associated immune dysregulation is characterized by the parallel occurrence of hyper-inflammatory reactions and immunosuppression (2–4). The term immunosuppression describes a heterogeneous scenario of an insufficient immune response to invading pathogens, which may affect both innate and adaptive immunity (5). Lipopolysaccharide (LPS), a membrane component of Gram-negative bacteria induces a special form of innate immunosuppression known as endotoxin tolerance (ET) characterized by a dampened response to ensuing LPS challenges. ET recapitulates several key features of immunosuppression as found in patients with sepsis (5, 6), and is therefore frequently assessed as a surrogate of the patient´s immune competence.

The innate immune system comprises several families of so-called pattern recognition receptors (PRR), that kick-start inflammatory signaling in response to selective pathogen-associated molecular patterns (PAMPs), such as LPS (7). Although individual PRR-dependent pathways can be compromised during immunosuppression (8, 9), the host cells are equipped with numerous other PRRs and effectors for surveillance of microbial presence (7). Inflammasomes are one class of cytosolic PRRs, constituting an important part of the innate immune arsenal. Inflammasome activation involves the assembly and activation of a multiprotein complex in response to damage-associated molecular patterns (DAMPs) or PAMPs exposed to the host cell surface or interior. Intracellular recognition of PAMPs can spark two different inflammasome pathways, the canonical and non-canonical inflammasome. Activation of the former is triggered through NOD-like receptor (NLR) or PYHIN family members, leading to caspase-1 activation, maturation of IL-1β, IL-18 (10–12). By contrast, activation of the non-canonical inflammasome is orchestrated by inflammatory caspases, caspase-4 and caspase-5 in humans and caspase-11 in mice (12–16), in response to the direct activation of these caspases by PAMPs (17–21). In addition, active inflammatory caspases 1, 4, 5 and 11 trigger pyroptosis by cleaving the linker between the N- and C-terminal domains of GSDMD, yielding a cytotoxic N-terminal (p30) fragment when released from the C-terminal (p20) repressor fragment. The N-terminal GSDMD fragment induces the formation of a large plasma membrane pore, culminating in pyroptotic cell death to eliminate intracellular bacteria (10, 22, 23).

Although caspase-4 and -5 are highly related, they exhibit differences in their regulation (24, 25). In particular, caspase-4 is constitutively expressed in monocytes/macrophages (16, 26–28), whereas caspase-5 and caspase-11 are induced through TLR/TRIF activation during inflammation (26, 28–33). In rodents, caspase-11 is transcriptionally induced and post-transcriptionally activated by IFN signaling (30, 32, 34–38). In humans, interferon regulatory factors 1 (IRF1) and 2 (IRF2) transcriptionally upregulate caspase-4 and GSDMD (39, 40). Consistent with this scenario, genetic deletion of non-canonical inflammasome components in mice, including *Casp11*, *Gsdmd*, *Irf1* or *Irf2* markedly attenuates the response to cytosolic PAMPs and protects against LPS-induced endotoxic shock (10, 13, 14, 30, 32, 34, 41, 42).

In addition to variable upstream regulation, caspase-4 and -5 also exhibit functional differences in their downstream output. Thus, caspase-4 and -11, but not caspase-5, induce the release of high mobility group box 1 (HMGB1) (16, 18, 43), while caspase-5 and -11 preferentially trigger cleavage and release of Interleukin-1α (IL-1α) (18, 25). However, despite the reported differences, it is often tacitly assumed that caspase-4, -5 and -11 play largely redundant roles in inflammatory cell death and cytokine release. This notion is likely fueled by early findings that both caspase-4 and caspase-5 can rescue LPS-induced pyroptosis in murine caspase-11 deficient macrophages (16).

In the context of severe infections in humans, the inflammasome executor caspase-1 is known to play a critical role in pyroptotic cell death and in the pathogenesis of sepsis (13, 14, 42, 44). In contrast, the role and regulation of the non-canonical inflammasome receptors caspase-4 and caspase-5 is only rudimentarily understood. In this study, we have explored the regulation of non-canonical inflammasome constituents *CASP4*, *CASP5* and *GSDMD* during sepsis and acute-on-chronic liver failure (ACLF)-associated immunosuppression. We exploited the expression profile of HLA-DRA and miR-222, a newly described marker of innate immunosuppression (6), as surrogates of immune competence for stratification of patients with sepsis or ACLF (4, 5, 45–48). Our data reveal differential regulation of *CASP4* and *CASP5* in patients with severe disease courses characterized by immunosuppression, organ damage and higher GSDMD activity, supporting a critical role of the non-canonical inflammasome pathway during the acquisition of immunosuppression.

## Materials and methods

### Patient recruitment, clinical characteristics and sample collection

**Figure 1:**
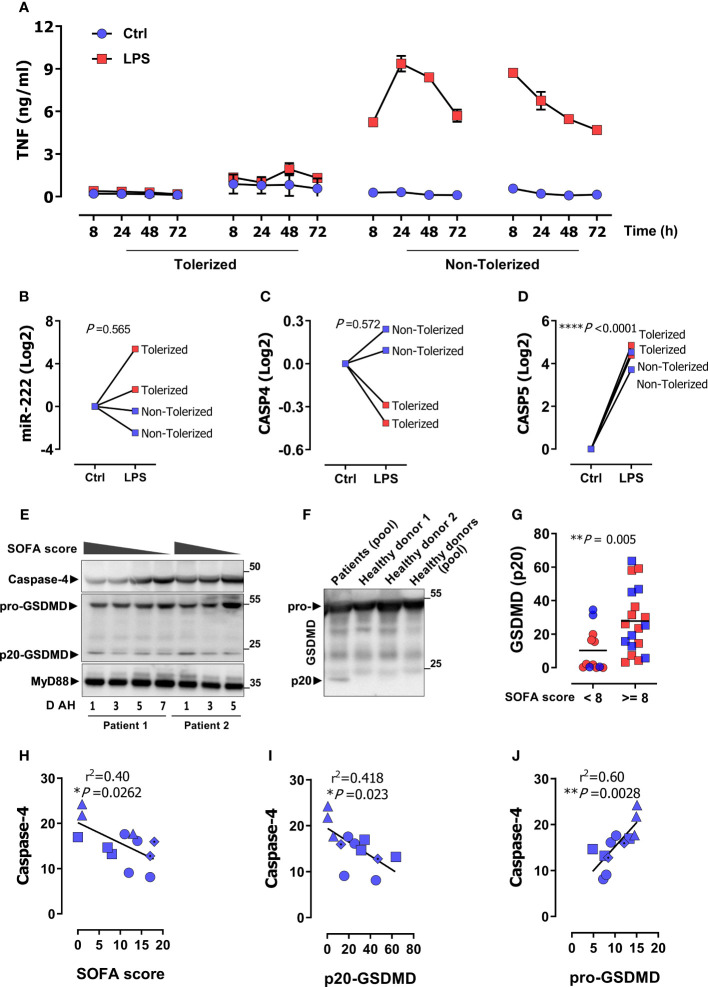
*CASP4* is suppressed in monocytes from patients with sepsis-associated immune suppression. **(A)** Peripheral monocytes from 4 sepsis patients were stimulated as indicated with LPS for 72 h and TNF levels were measured in supernatants by ELISA over the indicated time. Two patients showed a missing TNF response and were classified as tolerized/immunosuppressed. **(B–D)** Gene expression of miR-222 **(B)**, *CASP4* **(C)**, and *CASP5* **(D)** after 72 h LPS stimulation of monocytes from the same patients as in **(A)**. *P* values from unpaired two-tailed *t-*test. **(E)** A representative immunoblot of the protein amount of caspase-4, GSDMD and MyD88 (loading control) in serum of two sepsis patients along with SOFA score and days after hospitalization (D AH). **(F)** Representative immunoblot of the protein amount of GSDMD in pooled serum of the sepsis patients from panel **(E)** and individual healthy donors or human serum pool (P30-2901, PAN-Biotech). **(G)** p20-GSDMD protein levels in enrolled sepsis patients with gram negative (blue symbols; clinical data in **Supplementary Table 2**) or gram-positive infection (red symbols; patient data in **Supplementary Table 3**). Patients are stratified for higher/lower than a SOFA score of 8 as indicated. *P* values from two-tailed Mann-Whitney U test. **(H–J)** Linear correlation analysis of caspase-4 protein expression with SOFA score **(H)** p20-GSDMD **(I)** and pro-GSDMD **(J)**. Each data symbol represents a different patient with corresponding SOFA scores on different days of hospitalization (D AH). *P* value from two-tailed Pearson´s correlation. *P < 0.05, **P < 0.01, ***P < 0.001, ****P < 0.0001, r^2^ value from the Pearson correlation coefficient.

Human peripheral blood cells (PBMCs) were obtained after informed consent from patients or legal representatives. Patient consent guidelines, isolation and characterization of human immune cells and the use of clinical data was approved by the internal review board of the ethics committee of the Jena University Hospital. ACLF cohort: Ethics vote no. 3683-02/3. Sepsis cohorts: Ethics votes nos. 2160-11/07, 2712-12/09 and 3824-11/12. All involved procedures were in accordance with the principles of the Declaration of Helsinki (49). The clinical characteristics of sepsis patients enrolled for the experiments shown in **Figures 1A–D** and **Figures 1E, F, H, I, J** are summarized in **Supplementary Tables 1**, **2**, respectively. The clinical data of patients enrolled for **Figure 1G** are presented separately for Gram-negative (**Supplementary Table 2**) and Gram-positive sepsis (**Supplementary Table 3**). All individuals with sepsis fulfilled the Sepsis-3 definition. First blood sample was drawn within 24 h after sepsis symptoms emerged. Patients for cohorts of acute decompensation of chronic liver disease and suspected bacterial infection were recruited from hospitalized patients at boards of the University Hospital, Jena, Germany. Baseline characteristics and outcome of these patients with decompensated liver disease in the absence or presence of multiple organ failure syndrome (according to the EASL CLIF-C criteria for acute-on-chronic liver failure) are given in **Supplementary Table 4**. Clinical scores such as model for end-stage liver disease scores (MELD), bacterial culture count, protein analysis, blood count and serum levels of C-reactive protein and creatinine were obtained from routine laboratory analysis.

### Blood, PBMC and CD14^+^ monocyte isolation and processing

PBMCs were isolated from whole blood of patients and healthy volunteers by density gradient centrifugation using Biocoll (Cat#L6715,Merck) according to the manufacturer’s protocol. PBMCs and CD14^+^ monocytes of patients with acute decompensation of liver cirrhosis with ascites were purified using positive magnetic-activated cell sorting (Miltenyi Biotec, Germany) and stored at -80°C prior to use. Monocytes derived from whole blood of patients with sepsis (**Supplementary Table 1**) were isolated by density gradient centrifugation using Biocoll, washed in PBS and cultured in DMEM supplemented with 10 µg/ml ciprofloxacin and 10% human serum overnight. Next day, cells were washed twice with pre-warmed PBS and stimulated or not with 1 µg/ml LPS (Cat#tlrl-3pelps; InvivoGen) for 72 h. Supernatant and cells were harvested separately at different time points as indicated. Monocytes of healthy volunteers were differentiated into macrophages by culturing in DMEM medium supplemented with 10% FCS, 10 µg/ml ciprofloxacin and 10 ng/ml recombinant human M-CSF (Cat#574806, Biolegend) for 5 days. On day 6 cells were washed and stimulated with 50 ng/ml LPS alone or in combination with 100 ng/ml IFN-γ (Cat#570206, Biolegend) from 8 to 48 hours. Cells were detached using Trypsin-EDTA 0.25% (ThermoFisher) for FACS analysis or processed for real time PCR as described below. Supernatants were stored at -80 C° upon harvest for TNF measurements. To activate the non-canonical inflammasome, MDMs treated or not with LPS, IFN-γ or LPS+IFN-γ for 48h were transfected with LPS using lipofectamine-2000 (Cat#11668, Thermo Fisher) for 18h as previously described (50).

### RNA extraction, reverse transcription, and qPCR

Total RNA was extracted from samples using miRNeasy Mini Kit (Cat#217084, Qiagen). For reverse transcription of miRNAs to cDNA, the Universal cDNA Synthesis Kit (Cat#339340, Qiagen) was used. Real time PCR of miRNA was performed by the miRCURY LNA miRNA PCR Assay (Cat#339345, Qiagen) using locked nucleic acid primers (Qiagen). Baseline expression of the specific miRNA genes was normalized to small nuclear RNA U6 as an internal control gene. For mRNA-expression, approximately 0.5 μg of RNA was reverse transcribed using the RevertAid First Strand cDNA Synthesis Kit (Cat#K1621, Thermo Fisher). Genes of interest were amplified with specific primers using SybrGreen kit (Cat#331416, Biozym) according to the manufacturer’s instructions. mRNA expression profiling was performed using specific primer pairs and normalized to *GAPDH* as a reference gene. The primers used are given in **Supplementary Table 5**.

### Cytokine measurement and cytotoxicity assays

TNF was measured using the human TNF ELISA Set (Cat#DY210-05, R&D Systems) according to the manufacturer’s instructions. For analysis of pyroptotic cell death, lactate dehydrogenase (LDH) release was measured in cell-free culture supernatants by LDH-assay (Cat#MK401, Takara Clontech) according to manufacturer´s instructions.

### Immunoblotting

Protein concentration in serum and cell lysates was determined via BCA assay. Isolation of protein from supernatants and cell lysates and Western blotting was performed as previously described (50) using the following antibodies: MyD88 (E-11) Maus mAb, (Cat#sc-74532, SantaCruz), caspase-4 Rabbit pAb (Cat#4450; Cell Signaling Technology), IRF-1 (D5E4) XP^®^ Rabbit mAb (Cat#8478, Cell Signaling Technology), β-Actin (13E5) Rabbit mAb (HRP Conjugate) (Cat#5125, Cell Signaling Technology). GSDMD Rabbit pAb (Cat#20770-1-AP, Proteintech) was used to detect full-length GSDMD and p20-GSDMD in serum; GSDMDC1 Rabbit pAb (Cat#NBP2-33422, Novus Biologicals) was used to detect full-length GSDMD and p30-GSDMD in cell supernatants and lysates. Goat anti-mouse IgG (H+L)-HRP (Cat#074-1806, KPL), Goat anti-rabbit IgG (H+L)-HRP (Cat#074-1506, KPL). Proteins were visualized using ECL-substrate (Cat#541004, Biozym) in a GBOX-Chemi-XX6 gel documentation system (Syngene). Acquisition and densitometric analysis of relative fold differences in caspase-4 protein expression was performed using ImageJ software. Band intensity was determined in the caspase-4 band (~45 kDa) or GSDMD (~20kDa) as region of interest and expressed in arbitrary units.

### Flow cytometry

For surface marker staining of MDM, cells were blocked with FcR blocking reagent (Cat#130-059-901, Miltenyi Biotec) for 20min, then incubated with FITC-anti-human HLA-DR, Mouse IgG2a antibody, clone L243 (Cat#307604, Biolegend) or FITC- mouse IgG2a isotype control clone MOPC-173 (Cat#400209, Biolegend) for 1h. Samples were washed extensively with sterile FACS buffer (PBS, 2% FCS, 2 mM EDTA) then analyzed using CytExpert (Beckman). Cells were gated on singlets. Data were analyzed using FlowJo software (BD).

### Methylation analysis

400 ng genomic DNA from PBMCs was converted into bisulfite-treated bsDNA using EZ DNA Methylation-Gold™ (Cat#D5005, Zymo Research) according to the instruction manual. Identically treated unmethylated DNA (Cat#59665, Qiagen) served as negative control. Positive control sample was created by *in-vitro* methylation using the CpG-Methyltransferase (*M.SssI*) (Cat#M0226S, New England Biolabs). A serial dilution using *in vitro* methylated and unmethylated control bsDNA was created to generate standard samples of 0%, 3%, 6%, 12%, 24% and 100% methylation level. Samples and standards were amplified with bisulfite-specific primers (F: TTTTTTGATAATGAGTTTGGAAT; R: ACCTACCATAAAAAACAACCTC) targeting a region of the *CASP4* gene (Chr11:104968517-104968678). The PCR was conducted using the SybrGreen MasterMix (Cat#4913914001, Roche) with 0.5 µM of each primer and 25 ng bsDNA in a Mastercycler Gradient (Eppendorf). After clean-up with the innuPREP DOUBLEpure kit (Cat#845-KS-5050250, Analytik Jena), PCR products were sequenced by Sanger sequencing (Microsynth). Sequences were analyzed by BLAST alignments against the unmethylated reference sequence (NCBI) and by visual inspection of sequence profiles.

### Statistical analysis

Statistical analysis was performed using GraphPad prism version 6.0 (GraphPad Software, La Jolla, CA, USA) as indicated in the respective figure legends. Differences between cirrhosis patients with and without ACLF were analyzed by unpaired two-tailed *t*-test for parametric after Log2 transformation to reach normal distribution. Two-tailed Mann–Whitney U test was used for comparing unpaired, nonparametric data. Horizontal lines represent mean values. Correlations were analyzed using Pearson´s test representing r^2^ or linear regression. The values obtained for each individual donor were paired and a paired two-tailed *t*-test was used. *P* values are from two-tailed Pearson´s correlation of parametric regression analysis. Each data marker represents an individual patient. Data generated from *in vitro* experiments are presented as mean ± SEM. For all tests, *P* < 0.05, **P < 0.01, ***P < 0.001, ****P < 0.0001 was considered significant or ns as not significant.

## Results

### Down-regulation of *CASP4*, not *CASP5*, is associated with sepsis-induced immunosuppression

Patients with sepsis often exhibit signs of immunosuppression, including a dampened response of monocytes to LPS, a condition termed ET (51, 52). To understand the role played by the non-canonical inflammasome in this context, we examined *CASP4* and *CASP5* expression in monocytes from patients with sepsis and organ damage who did or did not exhibit ET. **Supplementary Table 1** shows the clinical characteristics of the enrolled patients. Consistent with previous reports (48, 51–53), some patients exhibited diminished LPS-induced tumor necrosis factor (TNF) release from monocytes, the hallmark of ET (**Figure 1A**). We refer to this state as `tolerized´ from here on. We have previously shown that LPS-dependent induction of the microRNA miR-222 discriminates between tolerized and non-tolerized patients (6). As shown in **Figure 1B**, the pattern of LPS-dependent miR-222 induction matched the TNF secretion profile, as monocytes from the tolerized patients exhibited higher miR-222 levels. Besides directly binding and activating *CASP4*/*CASP5*, LPS also induces expression of *CASP4* and *CASP5* in monocytes, a crucial priming step of the inflammasome (54). Remarkably, we observed a dichotomous response of *CASP4* to chronic LPS exposure, as LPS down-regulated *CASP4* in monocytes of tolerized patients while it upregulated *CASP4* in the non-tolerized patients (**Figure 1C**). In contrast, LPS stimulated *CASP5* expression in all patients (**Figure 1D**). In line with this transcriptional profile, caspase-4 protein levels in serum from patients with Gram-negative sepsis (**Supplementary Table 2**) inversely correlated with disease severity SOFA score (**Figures 1E, H**). We concluded that *CASP4* and *CASP5* were differentially regulated during the acquisition of a state of immunosuppression and that caspase-4 down-regulation was specifically associated to disease severity and immunosuppression. Activation of inflammatory caspases-4/5 results in cleavage of GSDMD into N-terminal (p30) and C-terminal (p20) fragments to induce pyroptotic cell death. Interestingly, patients with sepsis showed a prominent accumulation of the p20-GSDMD form (**Figure 1E**). To ascertain specificity of the detected p20 band, serum samples from healthy donors were loaded next to a pool of sepsis patients. GSDMD (p20) was detected only in patients, but not in healthy donors (**Figure 1F**). Furthermore, a cut-off value of ≥ 8 SOFA score was chosen to distinguish patients with a high disease severity. As shown in **Figure 1G**, p20-GSDMD levels were significantly higher in patients with a higher SOFA score. In addition, p20-GSDMD was negatively associated with caspase-4 expression (**Figure 1I**), suggesting less activity/expression of caspase-4 during immunosuppression-associated inflammasome activation and pyroptosis. In contrast, levels of the pro-GSDMD precursor form correlate positively with caspase-4 (**Figure 1J**), consistent with the notion that *GSDMD* and *CASP4* are jointly regulated by the transcription factors IRF1 and IRF2 (39, 40).

### Caspase-4 and caspase-5 genes are differentially regulated during immunosuppression-associated organ damage

**Figure 2:**
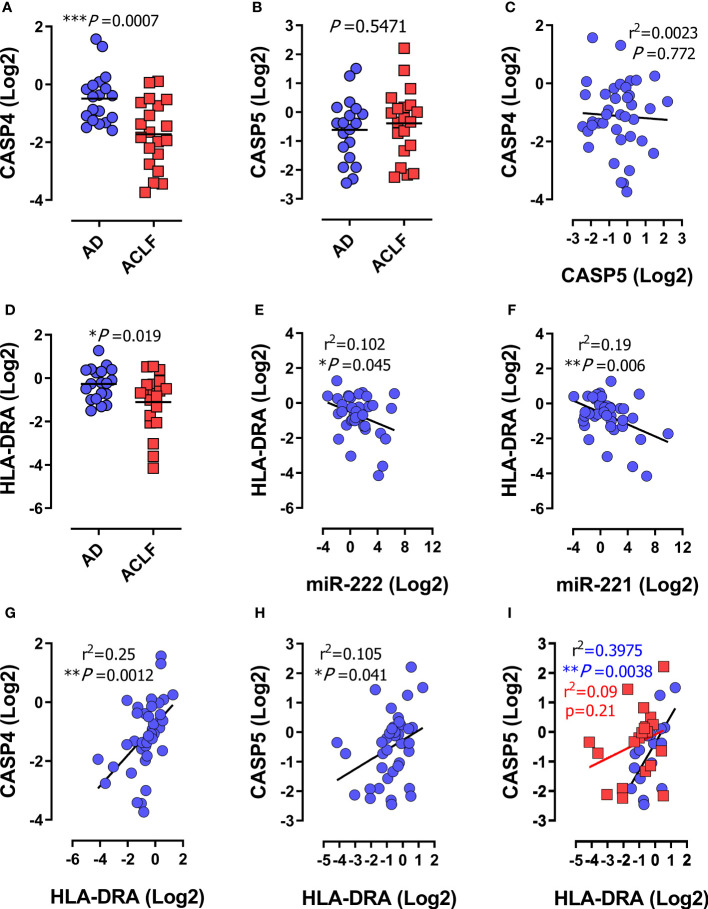
Human *CASP4* and *CASP5* are differentially regulated during inflammatory organ failure in patients with decompensated cirrhosis. **(A–I)** Expression analysis of the indicated genes in peripheral blood mononuclear cells (PBMCs) from patients with acute decompensation of cirrhosis (n=39). **(A–D)** mRNA expression of *CASP4* **(A)** *CASP5* **(B)** and *HLA-DRA* **(D)** in PBMCs from patients with acute decompensation of cirrhosis stratified for acute decompensation without ACLF (AD; n=19) or acute decompensation with ACLF (ACLF; n=20). **(C)** Correlation of mRNA expression of *CASP4* and *CASP5*. **(E–H)** Correlation of *HLA-DRA* with miR-222 **(E)**, miR-221 **(F)**, *CASP4* **(G)** or *CASP5* **(H)**. **(I)** Correlation of *HLA-DRA* expression with *CASP5* stratified for acute decompensation (AD) without ACLF (blue dots) versus ACLF (red dots). **(A, B, D)**: *P* values from two-tailed *t*-test. Horizontal lines represent mean values. **(C, D–I)**: *P* and r^2^ values from two-tailed Pearson´s correlation of parametric regression analysis. Each data marker represents an individual patient. * *P* < 0.05, ** *P* < 0.01, ***P < 0.001.

To understand if differential regulation of *CASP4 versus CASP5* was specific to Gram-negative sepsis or general to severe infections with immunosuppression, we studied a cohort of patients with liver cirrhosis and acute decompensation and ascites. Patients were stratified for presence of acute-on-chronic liver failure (ACLF) according to the criteria of the European Foundation for the study of chronic liver failure (47) (**Supplementary Table 4**). ACLF is an inflammatory syndrome of multiple organ failure (MOF) in patients with pre-existing liver disease often precipitated by inflammatory events such as bacterial infections and sharing many characteristics with sepsis (55). mRNA analysis of Peripheral Blood Mononuclear Cells (PBMCs) revealed that *CASP4* gene expression was suppressed specifically in patients with ACLF (**Figure 2A**), while *CASP5* expression was unaltered and independent of ACLF (**Figure 2B**). Consistently, no linear correlation was observed between *CASP4* and *CASP5* (**Figure 2C**). Analogous findings were obtained from purified CD14^+^ monocytes (**Supplementary Figures S1A–C**), pointing to this leukocyte type as a major involved cellular player. Indeed, we observed a significant increase in caspase-5 in the CD14-enriched population (**Figure S1B**), which was not apparent in PBMCs (**Figure 2B**), suggesting a specific regulatory pattern of caspase-5 in CD14^+^ monocytes (28). Regardless of the underlying mechanisms, the change in caspase-5 was invariably in the opposite direction to that of caspase-4. To determine whether ACLF patients did indeed suffer from immunosuppression, we measured the expression of miR-222, miR-221 (6), and *HLA-DRA*, which encodes the alpha subunit of HLA-DR and is routinely assessed as biomarker of immunosuppression in sepsis (2, 4, 5, 46, 48, 57) and cirrhosis (45). This analysis confirmed the presence of immunosuppression in the ACLF patient cohort (**Figures 2D–F**). *HLA-DRA* correlated inversely with miR-222 and miR-221 expression (**Figures 2E, F**), supporting that miR-222/221 and *HLA-DRA* do both reliably discriminate patients with immunosuppression. Furthermore, *CASP4* and *HLA-DRA* were positively correlated in PBMCs (**Figure 2G**) and CD14^+^ monocytes (**Supplementary Figure S1D**), confirming the association between low *CASP4* levels and immunosuppression. Interestingly, although *CASP5* expression correlated with *HLA-DRA* in the whole cohort of patients (**Figure 2H**), this link was lost in the subgroup of patients with ACLF (**Figure 2I**). Virtually the same correlations were obtained in CD14^+^ monocytes (**Supplementary Figures S1D, G**). These data indicated that *CASP4* but not *CASP5* is selectively downregulated in myeloid cells during immunosuppression associated to severe disease courses with MOF.

### Suppressed *CASP4* and *GSDMD* expression due to low *IRF2* and *IRF1* activity during immunosuppression-associated organ damage

**Figure 3:**
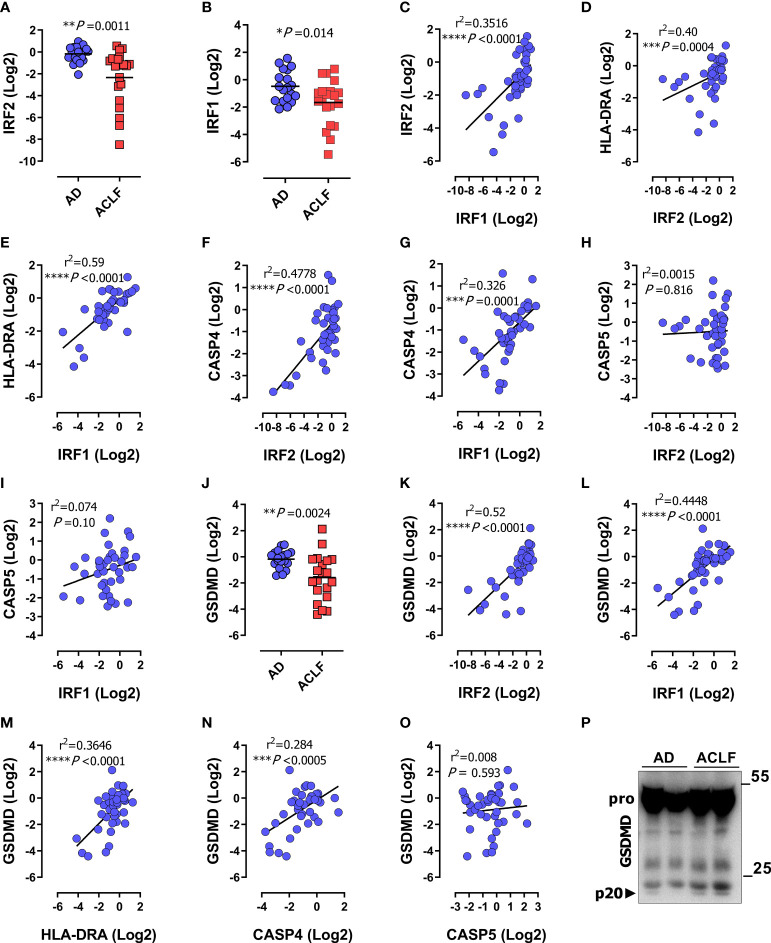
*IRF1* and *IRF2* are repressed during immunosuppression-associated organ failure in patients with decompensated cirrhosis. Data were obtained as described in **Figure 1**. **(A, B)** mRNA expression of *IRF2* **(A)**, and *IRF1* **(B)** in PBMCs of patients with acute decompensation (AD; n=19) or acute decompensation and ACLF (ACLF; n=20). **(C)** Correlation of mRNA expression of *IRF1* and *IRF2*. **(D, E)** Correlation of *HLA-DRA* mRNA expression in patient PBMCs with *IRF2* **(D)**, and *IRF1* **(E)**. **(F, G)** Correlation of mRNA expression of *CASP4* with *IRF2* **(F)** and *IRF1* **(G)**. **(H, I)** Correlation of mRNA expression of *CASP5* with *IRF2* **(H)** and *IRF1* **(I)**. **(J)** *GSDMD* mRNA expression in PBMCs of patients with acute decompensation without ACLF (AD; n=19) or with ACLF (n=20) **(J)** *P* values determined by unpaired two-tailed t-test, horizontal lines represent mean values. **(K–O)** Correlation of mRNA expression of *GSDMD* with *IRF2* **(K)**, *IRF1* **(L)**, *HLA-DRA* **(M)**, *CASP4* **(N)** and *CASP5* **(O)**. **(P)** Immunoblot of the protein amount of GSDMD and p20-GSDMD (p20) in serum of patients with AD or ACLF. *P* and r^2^ values from two-tailed Pearson´s correlation of parametric regression analysis *P < 0.05, **P < 0.01, ***P < 0.001, ****P < 0.0001. Each data marker represents an individual patient.

The transcription factors *IRF1* and *IRF2* mediate non-canonical inflammasome activation by driving the expression of *CASP4* and *GSDMD* genes at steady state and during infection (39, 40). To understand the mechanisms of *CASP4* regulation during immunosuppression, we examined the methylation pattern of the *CASP4* promoter. First, we confirmed the presence of IRF1 and IRF2 recognition sequences in the *CASP4* promoter region using the Pscan algorithm (58) (**Supplementary Figure S2A**). The *CASP4* promoter sequences recognized by IRF2 and IRF1 appeared to be palindromic (**Supplementary Figure S2B**) and included three putative methylation-sensitive CpG motifs: cg05618647, cg16669455 and cg16315582 (**Supplementary Figure S2A**). DNA methylation was analyzed in PBMCs of healthy donors (n = 5), patients with cirrhosis and ACLF (n = 5), and patients with cirrhosis without ACLF (n = 5). Artificial dilution series validated a detection limit of 10% methylation for Sanger sequencing of bulk PCR products. None of the analyzed clinical PBMC samples showed detectable methylation (exemplary data shown in **Supplementary Figure S2**). These data did not support a major contribution of promoter methylation to the reduced *CASP4* expression in patients with ACLF. Instead, we detected downregulation of *IRF1* and *IRF2* in patients with ACLF (**Figures 3A, B**). Accordingly, IRF1 and IRF2 correlated across the entire patient cohort with each other (**Figure 3C**) and with *HLA-DRA* (**Figures 3D, E**), indicating that *IRF1/IRF2* levels depended on the immune status. Of note, the expression of IRF1 and IRF2 positively correlated with *CASP4* (**Figures 3F, G**) but not *CASP5* (**Figures 3H, I**), supporting the notion that attenuated expression of IRF1/IRF2 mediates downregulation of *CASP4* in immunosuppressed patients. In addition, the expression of *GSDMD* was suppressed in patients with ACLF (**Figures 3J**) correlating with the expression of *IRF2*, *IRF1*, *HLA-DRA* and *CASP4* (**Figures 3K–N**) but not *CASP5* (**Figure 3O**). Analogous expression and correlation profiles in CD14^+^ monocytes corroborated the findings obtained in PBMCs (**Supplementary Figures S1E–I, J–L**).

To determine the extent to which disease severity, as reflected by MOF, truly correlated with the degree of immunosuppression in ACLF patients, we tested for correlation between HLA-DRA, as an immunosuppression marker, and IRF2 and IRF1 as *CASP4* regulators with the disease severity score MELD. *HLA-DRA*, *IRF2* and *IRF1* significantly correlated with MELD (**Supplementary Figures S3A–E**), as did the mononuclear expression profiles of *CASP4* and GSDMD. However, the MELD score did not correlate with *CASP5* (**Supplementary Figure S3F**). These data were consistent with the findings from **Figures 2A, B**, **3J** and confirmed that inflammatory organ damage is accompanied by the down-regulation of *CASP4* and *GSDMD*, but not *CASP5*. Since leukocytosis is a common hallmark of sepsis and severe inflammatory conditions, we also tested for correlation with white blood cell counts (WBCs) as a surrogate of systemic inflammation and found the same strong correlation with *HLA-DRA, IRF2*, *IRF1*, *CASP4*, and *GSDMD* (**Figures S3G–J, L**), but not *CASP5* (**Figure S3K**). Collectively, these data demonstrate that selective suppression of human inflammatory caspase-4 is related to disease severity and associated with downregulation of the transcriptional activators IRF1 and IRF2. Finally, analysis of cleaved GSDMD in sera from patients with acute decompensation (AD) without ACLF *versus* with ACLF recapitulated the pattern observed in patients with sepsis (compare **Figures 1E, F** above), i.e. an increased activation/cleavage of GSDMD in immunosuppressed ACLF patients, reflecting inflammasome activation, in a background of overall reduced transcriptional expression of GSDMD (**Figure 3P**).

### IFN-γ prevents ET-dependent *CASP4* suppression by upregulating *IRF2* and *IRF1* expression

**Figure 4:**
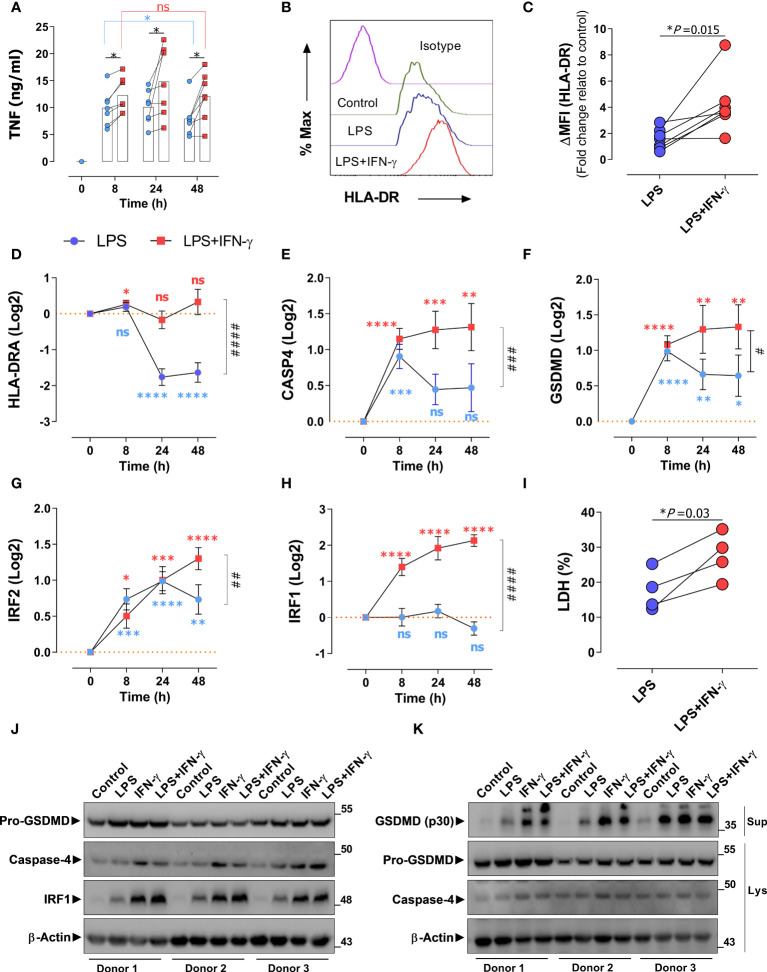
IFN-γ prevents endotoxin tolerance mediated *CASP4* suppression by upregulating *IRF2* and *IRF1* expression. **(A)** Monocyte-derived macrophages (MDM) from healthy donors (n=7) were stimulated with LPS alone (blue dots) or in combination with IFN-γ (red dots) and TNF production was analyzed via ELISA over the indicated time periods (hours). *Statistical difference between groups determined by (Wilcoxon test) for paired and nonparametric data. **(B)** A representative flow cytometry analysis of HLA-DR expression of donor MDMs after stimulation with LPS alone or with IFN-γ for 72 h. **(C)** Fold change relative to control (untreated) of median fluorescent intensity of HLA-DRA measured by flow cytometry on macrophages from healthy donors (n=7) treated as in panel **(A)** Each data point represents an individual donor. Statistical difference between groups determined by two tailed *t*-test for paired parametric values. **(D–H)** Expression of indicated genes in MDMs of healthy donors (n=7) in response to LPS alone (blue) or LPS with IFN-γ (56). **(I)** Cell death (%) measured by LDH-assay. Each linked data pair represents an individual donor. Statistical difference between groups determined by two tailed *t*-test for paired parametric values. **(J)** MDMs from healthy donors (n=3) were treated or not with LPS, IFN-γ or LPS+IFN-γ for 48h, protein amount of indicated proteins were detected by immunoblotting of cell lysates. **(K)** MDMs were treated as in A followed by transfection of LPS for 18h, protein amount of indicated proteins were detected by immunoblotting of cell supernatants (Sup) and lysates (Lys). *Significant to time-point 0 determined by Student’s t-test for unpaired parametric values. *P < 0.05, **P < 0.01, ***P < 0.001, ****P < 0.0001. ^#^ Significant to time point 48 h (LPS versus LPS+IFN-γ) determined by two-tailed *t*-test for paired parametric values. ^#^ *P* < 0.05, ^##^ *P* < 0.01, ^###^ *P* < 0.001, ^####^ *P* < 0.0001 determined by paired two-tailed t-test. ns, not significant. For all bar and line graphs, mean ± SEM is plotted.

Various studies have shown that ET can be prevented by treatment with IFN-γ (6, 59–61). To investigate the role of *CASP4* in IFN signaling in that context, we challenged human monocyte-derived macrophages (MDM) from healthy donors with LPS and IFN-γ in different combinations. Consistent with literature (6, 59), prolonged exposure of macrophages to LPS over days leads to a decrease in TNF production, reflecting the onset of ET (**Figure 4A**). Co-stimulation with IFN-γ boosted the LPS-induced TNF production, consistent with the notion that IFN-γ counteracts ET. Analogously, IFN-γ counteracted the LPS-induced down-regulation of HLA-DR in macrophages at the protein (**Figures 4B, C**) and transcriptional levels (**Figure 4D**). While the IFN-γ dependent rescue was consistently observed at the protein and gene level, we noticed a disparity when looking at the effect of LPS alone, as an apparent increase in HLA-DR surface protein (**Figure 4C**) was not reflected at the transcriptional level (**Figure 4D**). We hypothesize that different mechanisms of LPS-induced regulation of gene expression versus protein level may account for this discrepancy. Importantly, short-term LPS treatment induced *CASP4* expression in MDMs (**Figure 4E**), in line with previous reports (54) while prolonged exposure to LPS over 24h suppressed *CASP4*. Interestingly and analogous to *HLA-DRA* regulation, the reduction in *CASP4* expression was prevented by IFN-γ. The same response pattern was observed for GSDMD gene expression (**Figure 4F**), indicating that IFN-γ was able to rescue the expression of several components of the inflammasome in cells committed to tolerance. We further sought to clarify the association of *IRF1/IRF2* with *CASP4* and GDSMD expression in ET. Unlike *IRF1*, *IRF2* was upregulated upon acute stimulation with LPS (**Figures 4G, H**). However, *IRF2* levels saturated and started to decline 24 h post LPS challenge (**Figure 4G**), suggesting that *IRF2* was sensitive to tolerization. Simultaneous treatment with IFN-γ prevented the decline in *IRF2* levels and, strikingly, promoted a robust activation of IRF1 (**Figure 4H**). These data suggested that the IFN-γ dependent reversal of *CASP4* tolerization in human macrophages is mediated cooperatively via IRF1 and IRF2, consistent with findings by Benaoudia et al. (39). To confirm if the observed changes in gene transcription translated to protein levels and activity, we assessed protein expression of MDM cell extracts and supernatants (**Figures 4J, K**). These results confirmed the stimulatory effect of IFN-γ when added in conjunction with LPS at the level of caspase-4, IRF1 and GSDMD activation, as monitored by the levels of cleaved p30-GSDMD polypeptide in the MDM supernatant. We were unable to detect cleaved caspase-4, probably due to technical limitations of the antibodies used. Notably, IFN-γ alone showed a marked effect on protein levels of inflammasome components. Finally, we monitored cell death in the same MDM samples, as a readout of GSDMD activity and surrogate of inflammasome-dependent pyroptosis. As shown in **Figure 4I**, IFN-γ enhanced the low levels of pyroptotic cell death in LPS-treated MDMs, confirming that IFN-γ counteracts LPS-induced downregulation of the non-canonical inflammasome in a setting of immunosuppression. Taking everything together, these data show that *CASP4* behaves as a *bona-fide* ET-sensitive factor, suppressed by long-term LPS exposure and restored by IFN-γ.

## Discussion

An appropriate response to intracellular pathogens by the non-canonical inflammasome pathway is an important element of the host response to infection. Inflammasome activation requires first PRR activation by PAMPs to trigger synthesis of pathway components, followed by a second inflammatory trigger that leads to self-activation of caspase-1 and full-blown activation of the inflammasome. However, PRR-dependent priming can be impaired in ET or other settings of immunosuppression, affecting the activity and output of the inflammasome (44, 56). Indeed, mounting evidence indicates that deregulation of the inflammasome is involved in the pathogenesis of severe infections and sepsis (38, 62–64). Our analysis of human monocytes/PBMCs from two independent cohorts of critically ill patients with severe infection illustrates a specific down-regulation of caspase-4, not caspase-5, in sepsis and ACLF. Several features of this process are remarkable: First, down-regulation of caspase-4 was associated to the occurrence of immunosuppression and organ failure, indicating that the decrease in caspase-4 levels was primarily related to disease severity. In line with this notion, caspase-4 levels correlated with organ damage scores MELD and SOFA. Moreover, caspase-4 down-regulation was accompanied by a decline in GSDMD levels, a downstream executor of inflammasome-induced pyroptosis. This indicated that the decrease in caspase-4 was indeed associated with reduced inflammasome activity, suggesting that caspase-4 down-regulation was not coincidental but relevant to the innate host response. Second, the reduction of caspase-4 levels could be recapitulated *in vitro* by a prolonged challenge of macrophages with LPS. This observation illustrates that the downregulation of caspase-4 in sepsis and ACLF is not secondary to systemic complications, but likely represents an infection-proximal step in the monocytic host response. Third, caspase-4 downregulation was specific, as caspase-5 remained upregulated in patients with organ failure and immunosuppression, in line with previous reports (62, 63). Fourth, the decline in caspase-4 expression in tolerant macrophages was prevented by IFN-γ stimulation. Fifth, patients with signs of immunosuppression featured marked GSDMD activation, as illustrated by the accumulation of cleaved GSDMD-p20, in a background of low caspase-4 levels, suggesting that other inflammatory caspases, such as caspase-5, are likely to regulate GSDMD and pyroptosis during immunosuppression-associated organ damage (10, 23). Of note, in contrast to the simultaneously released N-terminal fragment p30, GSDMD-p20 does not have a pyroptotic function, but has instead been proposed to exert intracellular control of endocytic trafficking (65). Whether circulating GSDMD-p20 protein, as found in sepsis (**Figures 1F, G**), is beneficial or detrimental to organ function, perhaps by acting as a danger signal (DAMP), requires further investigation.

While studies in mice have shown that IFN-γ stimulates the non-canonical inflammasome by inducing caspase-11 (26), the regulation of the human orthologues caspase-4 and caspase-5 is far less clear. Previous reports have shown that activation of the non-canonical inflammasome requires IFN-signaling to initiate the production and assembly of multi-protein signaling platforms for the induction of pyroptosis (19, 26, 66, 67). Conversely, a suppressed macrophage response as found in ET can be restored through treatment with IFN-γ and is mediated by chromatin remodeling via stable STAT1 and IRF1 response-element occupancy and increased histone acetylation (6, 53, 59–61, 68, 69). Indeed, states of immunosuppression beyond ET are commonly associated with suppressed expression of interferon-responsive genes, leading to repressed autocrine and paracrine IFN-signaling with impaired activation of STAT and IRF transcription factors (6, 60, 70). Our findings are in line with this scenario, as the suppression of the non-canonical inflammasome components caspase-4 and GSDMD reported here correlated with repressed levels of its transcriptional regulators IRF2 and IRF1 (see **Figure 3**), which were both restored by treating tolerized macrophages with IFN-γ. Overall, our results are most readily explained by postulating impaired IFN/IRF1/IRF2 signaling in severely immunosuppressed patients as the cause of the decline in caspase-4 and GSDMD levels and thus inflammasome activity.

A further major hallmark of sepsis-associated immunosuppression is the apoptosis-related numerical and functional loss of immune cells including myeloid, T- and B-lymphocytes and NK cells (3, 5, 71). The drop in immunocyte number and function in turn is inseparably associated with the decreased expression and secretion of important immune regulators, prominently HLA-DR, IFN-γ and co-stimulatory molecule genes including CD4 and CD8 (2, 5, 53). Indeed, accumulating evidence implicates lymphocyte apoptosis, causing lymphopenia and reduced IFN-γ production, as a key kick-starting event in the immunosuppressive state of sepsis (72–74). One important consequence of Type I IFN and IFN-γ signaling is the promotion of pyroptosis through IFNAR1- and/or IFNGR1-dependent mechanisms initiated by PAMPs or pro-inflammatory cytokines. Consequently, diminished or defective IFN-signaling, as a result e.g. of reduced IFN-production in lymphopenia, may lead to impaired regulation of the non-canonical inflammasome (26, 34). Our present results strongly suggest that down-regulation of caspase-4 against a background of low IFN signaling, as prevalent in lymphopenia, may be a major cause of inflammasome dysfunction and innate immunosuppression in human severe infections.

In summary, our findings reveal an unexpected divergent regulation of caspase-4 and caspase-5 in severe inflammatory conditions. We identify *CASP4* as a *bona fide* marker of sepsis and ACLF disease severity and propose that it may perform better than currently used markers, given the strong and selective association of CASP4 levels with the most severe condition of immunosuppression and MOF. Caspase-4 may therefore not only prove to be a meaningful marker of sepsis severity, but its inactivation in sepsis is also likely to have important functional consequences, potentially compromising the immune response against intracellular bacteria and contributing to sepsis-related immunosuppression. Along these lines, CASP4 levels may also turn out an important determinant in guiding the use of therapeutic strategies targeting LPS or other Gram-negative PAMPs, such as extracorporeal endotoxin absorption, all of which have yielded disappointing results in the past (75, 76).

## Data availability statement

The raw data supporting the conclusions of this article will be made available by the authors, without undue reservation.

## Ethics statement

The studies involving humans were approved by Ethik-Kommission der Friedrich-Schiller-Universität Jena Bachstraße 18/Gebäude 1 07740 Jena Tel.: +49 3641 9-391191 Fax: +49 3641 9-391192. The studies were conducted in accordance with the local legislation and institutional requirements. The participants provided their written informed consent to participate in this study.

## Author contributions

MG performed most of the experiments: SD, MR, LR and BG carried out some experiments and contributed to sample preparation. NH performed DNA methylation analysis. MG, PR, FB, CS and TB organized and coordinated clinical trials and patient recruitment. MG, MB, TB and IR coordinated the study. MG and TB analyzed and processed data. MG and IR wrote the manuscript. All authors read, revised and approved the manuscript.
