# Supplementary material for: The non-canonical inflammasome activators Caspase-4 and Caspase-5 are differentially regulated during immunosuppression-associated organ damage
Source: Front Immunol. 2023 Dec 1;14:1239474. doi: 10.3389/fimmu.2023.1239474 (PMC10722270; doi:10.3389/fimmu.2023.1239474)
Supplement: Supplementary file 1 [file DataSheet_1.pdf]

Supplementary figures

The non-canonical inflammasome activators Caspase-4 and Caspase-5 are differentially regulated during immunosuppression-associated organ damage

Mohamed Ghait[1], Shivalee N. Duduskar[1], Michael Rooney[1,2], Norman Häfner[3], Laura Reng[1], Bianca Göhrig[1], Philipp A. Reuken[2] Frank Bloos[4], Michael Bauer[1,4], Christoph Sponholz[4], Tony Bruns[2,5 *], Ignacio Rubio[1,4 *]

[1]Integrated Research and Treatment Center, Center for Sepsis Control and Care, Jena University Hospital, Jena, Germany

[2]Department of Internal Medicine IV, Jena University Hospital, Jena, Germany

[3]Department of Gynecology, Jena University Hospital, Jena, Germany

[4]Department for Anesthesiology & Intensive Care Medicine, Jena University Hospital, Jena, Germany

[5]Department of Medicine III, University Hospital RWTH Aachen, Aachen, Germany
[*] These authors contributed equally.

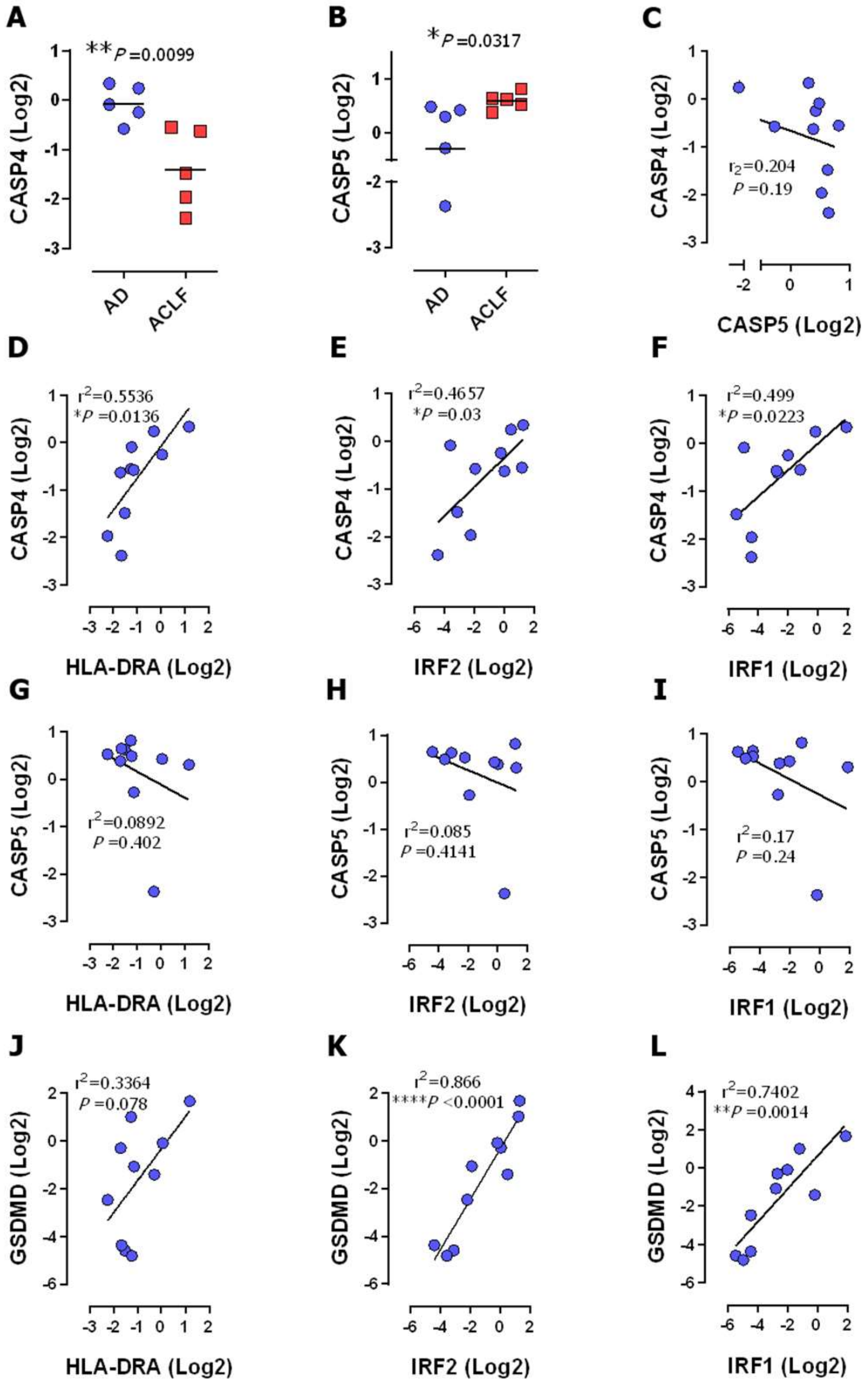

Supplementary Fig.S1. Human *CASP4* and *CASP5* are differentially regulated during innate immunosuppression state in CD14⁺ monocytes. (**A, B**) *CASP4* and *CASP5* Gene expression in $CD14^+$ monocytes of patients with chronic liver disease stratified for acute-on-chronic liver failure (ACLF) (+/- MOF) (n=10). *P* values, two-tailed *t*-test. Horizontal lines represent mean values. (**C**) Correlation analysis of mRNA expression of *CASP4* and *CASP5* in all patients. (**D-F**) Correlation of *CASP4* expression with *HLA-DRA*, *IRF2* and *IRF1*. (**G-I**) Correlation of *CASP5* expression with *HLA-DRA*, *IRF2* and *IRF1*. (**J-L**), Correlation analysis of *GSDMD* expression with *HLA-DRA, IRF2* and *IRF1*. Correlation coefficient (r^2) and *P* value from two-tailed Pearson´s correlation of parametric regression analysis. Each data marker represents an individual patient. *$P < 0.05$, **$P < 0.01$, ***$P < 0.001$, ****$P < 0.0001$.

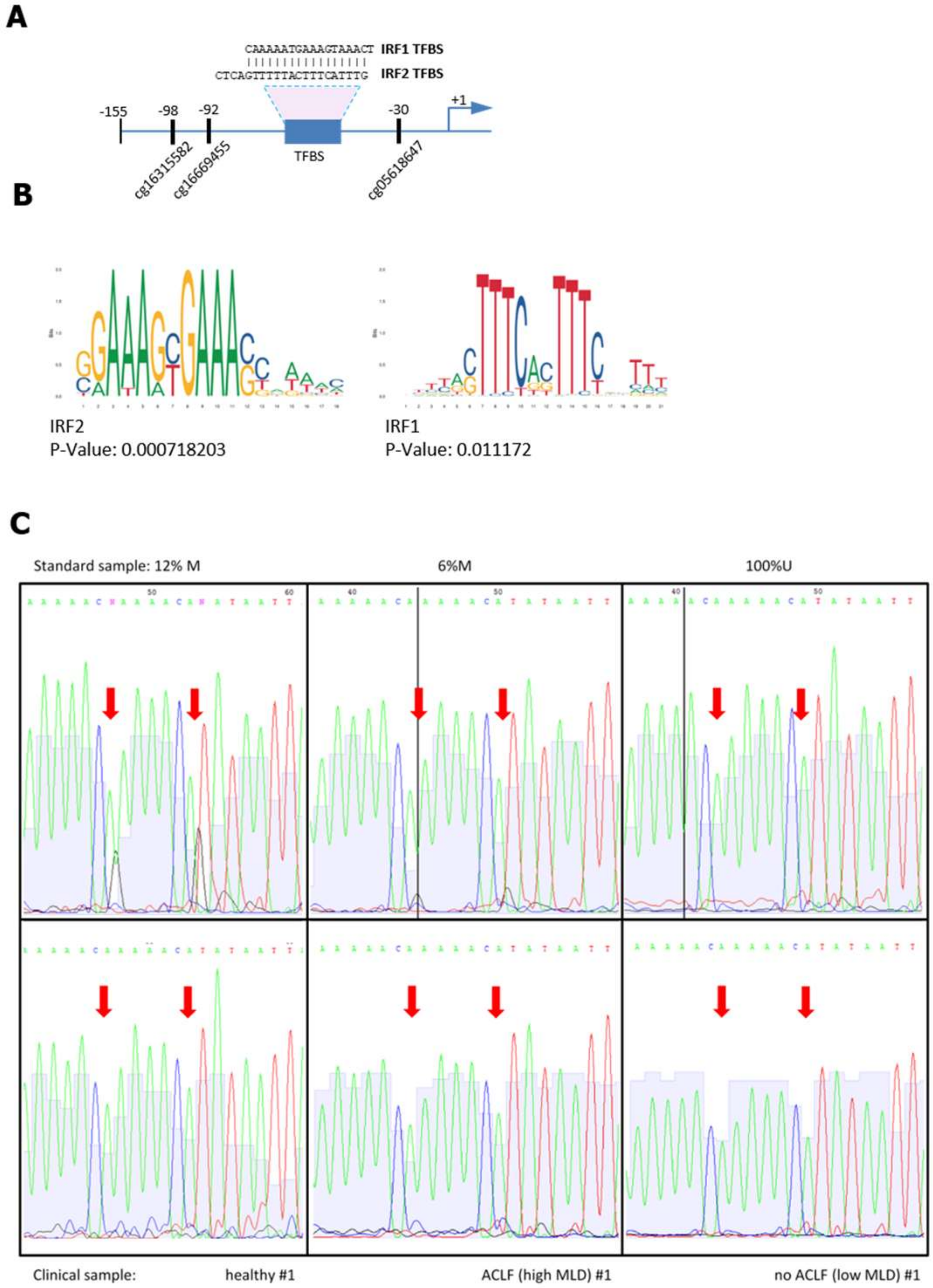

Supplementary Fig.S2. Analysis of IRF1 and IRF2 promoter methylation (A) CpG sites located in close proximity of IRF1 and IRF2 transcription factor binding sites (TFBS) of the *CASP4* gene. (**B**) Transcription factor binding motifs of IRF2 and IRF1 found by the Pscan algorithm in the *CASP4* promoter region. **(C)** Sanger sequencing profiles of the reverse strand from BS-PCR products. Shown are standards (upper panel: 12% methylation, 6% methylation, unmethylated DNA) and exemplarily samples from a healthy donor and patients without ACLF or with ACLF (lower panel). Importantly, none of the clinical samples showed a methylation intensity above the detection limit of 10% methylation. Red arrows mark the positions of CpGs. A methylated cytosine would result in a guanine on the reverse strand as seen in the standard samples (underrepresented guanine signal, black curve).

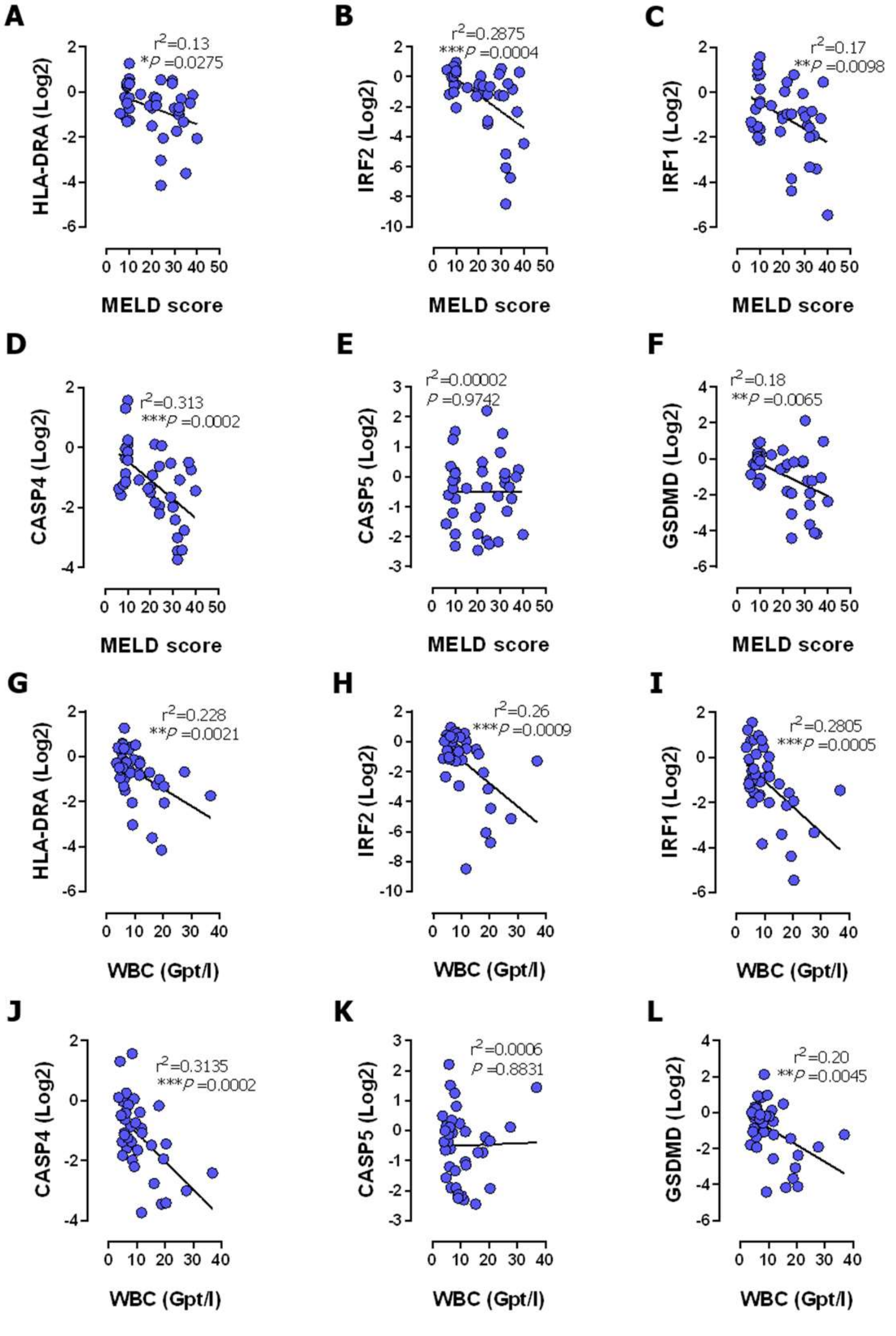

Supplementary Fig.S3. ***CASP4*** **but not** ***CASP5*** **expression correlates with surrogates of organ dysfunction in patients with decompensated cirrhosis**. Data were obtained as described in Fig. 2. (**A-F**) Correlation analysis of mRNA expression of the indicated genes in patient PBMCs with the MELD score**.** (**G-L**) Correlation analysis of mRNA expression of indicated genes in PBMCs with white blood cell counts (WBC). *P* and r^2 values from two-tailed Pearson´s correlation of parametric regression analysis **P* < 0.05, **P < 0.01, ****P* < 0.001, *****P* < 0.0001. Each data marker represents an individual patient.
