# Supplementary material for: The non-canonical inflammasome activators Caspase-4 and Caspase-5 are differentially regulated during immunosuppression-associated organ damage
Source: Front Immunol. 2023 Dec 1;14:1239474. doi: 10.3389/fimmu.2023.1239474 (PMC10722270; doi:10.3389/fimmu.2023.1239474)
Supplement: Supplementary file 2 [file Table_1.docx]

### Supplementary table 1: Characteristics of patients with sepsis analysed in Fig.1A-D.

|  | **Patient 1** | **Patient 2** | **Patient 3** | **Patient 4** |
| --- | --- | --- | --- | --- |
| **Age [years]** | 53 | 75 | 60 | 43 |
| **Gender** | male | female | male | male |
| **Cause of sepsis** | Pneumonia | Peritonitis | Pneumonia | Surgical site infection |
| **28-days mortality** | Alive | Alive | Alive | Alive |
