# Supplementary material for: The non-canonical inflammasome activators Caspase-4 and Caspase-5 are differentially regulated during immunosuppression-associated organ damage
Source: Front Immunol. 2023 Dec 1;14:1239474. doi: 10.3389/fimmu.2023.1239474 (PMC10722270; doi:10.3389/fimmu.2023.1239474)
Supplement: Supplementary file 3 [file Table_2.docx]

### Supplementary table 2: Characteristics of 4 patients with gram-negative sepsis related to Figs. 1E, F, H, I, J

| **Parameter** | **Mean or cases** | **Range or fraction** |
| --- | --- | --- |
| Age [years] | 67 | [84 - 49] |
| Gender, male | n = 3 | (75 %) |
| APACHE-II on admission | 27 | [15.0- 51.0] |
| SAPS-II on admission | 51 | [31.0 - 85.0] |
| 28-days mortality | n = 1 | (25 %) |
| **Site of infection** | | |
| Pneumonia | n=1 |  |
| Pneumonia, pacemaker-related infection | n=1 |  |
| Peritonitis due to sigma diverticulitis | n=1 |  |
| Surgical site infection | n=1 |  |
