# Supplementary material for: The non-canonical inflammasome activators Caspase-4 and Caspase-5 are differentially regulated during immunosuppression-associated organ damage
Source: Front Immunol. 2023 Dec 1;14:1239474. doi: 10.3389/fimmu.2023.1239474 (PMC10722270; doi:10.3389/fimmu.2023.1239474)
Supplement: Supplementary file 4 [file Table_3.docx]

### Supplementary table 3: Characteristics of 6 patients with gram-positive sepsis related to Fig.1G.

| **Parameter** | **Mean or average** | **Range or fraction** |
| --- | --- | --- |
| Age [years] | 67 | [84 - 49] |
| Gender, male | 4 | (66 %) |
| APACHE-II on admission | 25 | [20-35] |
| SAPS-II on admission | 53 | [29-80] |
| 28-days mortality | 4 | (66 %) |
| **Site of infection** | | |
| Lung (Pneumonia) | n=3 |  |
| Abdominal (Peritonitis) | n=1 |  |
| Skin (Surgical site infection) | n=1 |  |
| Blood (Cathether-related infection) | n=1 |  |
