# Supplementary material for: The non-canonical inflammasome activators Caspase-4 and Caspase-5 are differentially regulated during immunosuppression-associated organ damage
Source: Front Immunol. 2023 Dec 1;14:1239474. doi: 10.3389/fimmu.2023.1239474 (PMC10722270; doi:10.3389/fimmu.2023.1239474)
Supplement: Supplementary file 5 [file Table_4.docx]

### Supplementary table 4: Baseline characteristics of patients with decompensated liver disease in the absence or presence of multiple organ failure syndrome based on the EASL CLIF-C criteria for acute-on-chronic liver failure (ACLF).

|  | **Acute Decompensation without organ failure**  **N=20** | **Acute Decompensation with organ failure (ACLF)**  **N=19** | ***P* value** |
| --- | --- | --- | --- |
| Age (years) | 59 (52-66) | 55 (51-68) | 0.74 |
| Male sex | 14 (70%) | 16 (84%) | 0.45 |
| Alcoholic liver disease | 18 (90%) | 15 (79%) | 0.41 |
| Ascites | 20 (100%) | 19 (100%) | 1.00 |
| ACLF grade (1 / 2 / 3) | 0 / 0 / 0 | 9 (47%) /5 (26%) /5 (26%) | N/A |
| Infection  - None  - Peritonitis  - Urinary tract  - Pneumonia  - Other | - 17 (85%)  - 2 (10%)  - 1 (5%)  - 0  - 0 | - 9 (47%)  - 6 (32%)  - 2 (11%)  - 1 (5%)  - 1 (5%) | 0.02* |
| Creatinine (µmol/l) | 87 (74-108) | 236 (188-297) | <0.001 |
| Total bilirubin (µmol/l) | 16 (11-25) | 174 (44-423) | <0.001 |
| Model for end-stage liver disease (MELD) score | 10 (9-10) | 31 (24-34) | <0.001 |
| White blood cells (x10^3^/µl) | 6.4 (5.6-8.2) | 10.2 (8.0-19.4) | 0.02 |
| C-reactive protein (mg/l) | 18 (9-25) | 46 (24-118) | 0.002 |
| Liver transplant or death within 30 days | 1 (5%) | 10 (53%) | 0.001 |

Data are shown as frequencies or medians with interquartiles. N/A: not applicable. P values from Fisher’s exact test or Mann-Whitney U test as appropriate. *comparing infection versus no infection.
