# Supplementary material for: The non-canonical inflammasome activators Caspase-4 and Caspase-5 are differentially regulated during immunosuppression-associated organ damage
Source: Front Immunol. 2023 Dec 1;14:1239474. doi: 10.3389/fimmu.2023.1239474 (PMC10722270; doi:10.3389/fimmu.2023.1239474)
Supplement: Supplementary file 6 [file Table_5.docx]

**Supplementary table 5:** Primers used in this study.

| **Gene** | **Forward primer (5’-3’)** | **Reverse primer (5’-3)** |
| --- | --- | --- |
| *GAPDH* | AGGGCTGCTTTTAACTCTGGT | CCCCACTTGATTTTGGAGGGA |
| *CASP4* | AAGAGAAGCAACGTATGGCAGGAC | GGACAAAGCTTGAGGGCATCTGTA |
| *CASP5* | AGCATCCTTGGCACTCATCT | CCAGGACACGTTATGTGGTG |
| *HLA-DRA* | TTTCCGCAAGTTCCACTATCTCCC | AATAATGATGCCCACCAGACCCAC |
| *IRF1* | CTCCACCTCTGAAGCTACAA | TCCAGGTTCATTGAGTAGGT |
| *IRF2* | GGCTCAAGTGGCTTAACAA | CTGGTTGATGCTTTCCTGTAT |
| *GSDMD* | GTAGACTGGCCACATGGCTA | CTGGGTCTTGCTGGACGAGT |
